# Healthcare Infrastructure and Resource Barriers to Preventing Mother-to-Child Transmission of HIV in Ghana: Insights from a Qualitative Study

**DOI:** 10.3390/ijerph23030293

**Published:** 2026-02-27

**Authors:** Awinaba Amoah Adongo, Dominic Nabil Bodpii, Robert Kuchengye Mokulogo, Lumbwe Chola, James Akazili

**Affiliations:** 1 Department of Sociology and Social Work, Faculty of Social Sciences, Kwame Nkrumah University of Science and Technology, Kumasi A0K315, Ghana; 2 School of Business, University for Development Studies, Tamale TL1350, Ghana; dominic.bodpii@uds.edu.gh; 3 Department of Languages Education, School of Education and Life-Long Learning, Simon Diedong University of Business and Integrated Development Studies, Wa WA64, Ghana; rmokulogo@ubids.edu.gh; 4 Department of Health Management and Health Economics, Faculty of Medicine, University of Oslo, 0317 Oslo, Norway; lumbwe.chola@medisin.uio.no; 5 School of Public Health, C.K. Tedam University of Technology and Applied Sciences, Navrongo P. O. Box 24, Ghana; jakazili@cktutas.edu.gh

**Keywords:** healthcare access, health infrastructure, maternal health, barriers to care, patient experience

## Abstract

Background: The prevention of the mother-to-child transmission (PMTCT) of HIV is a vital strategy in reducing paediatric HIV infections. However, the delivery of PMTCT services is frequently impeded by resource constraints within the healthcare systems. This study investigates the systemic barriers affecting PMTCT implementation in Ghana and examines the disconnection between health policy design, priority setting, and on-the-ground realities. Methods: The study employed the qualitative approach using a case study research design. The purposive sampling technique was used in selecting the health facilities, with an in-depth interview guide used to solicit views from healthcare providers and mothers participating in PMTCT services. Braun and Clarke’s thematic analysis was employed in analysing the data on the perceptions of infrastructural and resource-related challenges affecting PMTCT services. Results: Participants identified several key barriers, including the absence of dedicated office spaces, a limited outpatient department (OPD) capacity, inadequate storage for antiretroviral therapy (ART) medications, and shortages of HIV-testing equipment affecting care delivery and access. These issues, alongside workforce limitations and supply chain disruptions, were found to significantly undermine the delivery and effectiveness of PMTCT services. Conclusions: The study underscores the need for context-aware health policy development. Effective priority setting and benefits package design must be informed by frontline insights, taking into account infrastructural deficits, human resource constraints, and systemic bottlenecks. Aligning national initiatives, such as the StEPS programme, with operational realities is essential for enhancing PMTCT outcomes.

## 1. Introduction

The transmission of HIV from mother to child (MTCT) accounts for 70–80% of paediatric HIV infections in Ghana [1]. The prevalence and incidence rates of MTCT stand at 2.4% and 15.6 per 100,000 live births, respectively [1,2,3]. This calls for interventions targeted at a drastic reduction in MTCT [3]. For that reason, the World Health Organization (WHO) promotes PMTCT strategies that include antiretroviral therapy (ART) for mothers during pregnancy, labour, and breastfeeding, which has proven to be highly cost-effective [4,5]. It is also one of the key essential interventions recommended for inclusion in health benefits packages in low-resource settings for mothers [6]. Nevertheless, systemic issues such as inadequate healthcare logistics to ensure access to PMTCT services continue to hinder effective PMTCT services in sub-Saharan Africa [7]. Many mothers do not receive sufficient care due to infrastructural inadequacies, including insufficient healthcare facilities [8,9]. Evidence suggests that the persistent lack of access to healthcare and logistics continues to affect access to critical PMTCT services in sub-Saharan Africa, increasing the risk of HIV transmission to infants [10]. This eventually prevents mothers from benefiting from these cost-effective interventions [10].

Although the integration of PMTCT services into the maternal and child health programmes in Ghana has led to improved outcomes, the priority, as envisioned by the Ministry of Health and Ghana Health Service, on access and coverage remains a challenge [5]. For instance, the Ghana AIDS Commission reported that only 62% of pregnant women living with HIV received ART in 2020 [11,12,13,14]. Thus, reference. Ref. [15] suggests that such systemic bottlenecks should be considered when designing health benefits packages for maternal and child health interventions, as they can significantly impact the accessibility, effectiveness, and sustainability of these programmes [13]. The reason is that the role of the healthcare infrastructure is crucial in enhancing PMTCT, especially in low- and middle-income jurisdictions where the availability of a standard healthcare infrastructure and medical supplies can influence the availability of trained medical personnel to deliver PMTCT services. Monyane and Rantao [14,15] associated inadequate healthcare facilities in low- and middle-income jurisdictions with poor maternal health outcomes, and called for the investment in a modern and standard healthcare infrastructure to support healthcare delivery for vulnerable populations [10].

There is substantial evidence that resource deficits continue to pose a significant barrier to effective healthcare delivery across many parts of Africa. For example, the World Health Organization (WHO) reports that sub-Saharan Africa carries 24% of the global disease burden, and, yet, has only 3% of the world’s health workforce and less than 1% of global health expenditures [14]. Infrastructure challenges are also pronounced; a 2018 cross-country assessment found that only 28% of health facilities in low-income African countries had access to reliable electricity, and fewer than 40% had basic water services. In the context of HIV care, studies have shown that many countries still experience critical shortages of antiretroviral therapy (ART), trained healthcare personnel, and diagnostic tools, particularly in rural settings [10]. These persistent deficits severely hinder the efforts to prevent the mother-to-child transmission (PMTCT) of HIV and compromise overall health outcomes for vulnerable populations.

This call is critical because inadequate facilities can lead to missed opportunities for ART initiation, and, thus, affecting the health outcome of the mother-to-child transmission of HIV [16]. It is well-established that infrastructural deficits are a major barrier to accessing HIV care in Africa [17]. This situation is worrying and requires empirical evidence to serve as a roadmap to addressing these challenges across low- and middle-income areas; yet, the existing literature emphasises the correlation between infrastructural deficits and adverse health outcomes, while neglecting the lived experience of PMTCT services among victims [18,19]. The sharing of such experiences on the effect of an inadequate healthcare infrastructure, including insufficient medical resources, hampers the effectiveness of PMTCT services. Although some studies have been carried out in this area, a qualitative inquiry to gain the lived experiences of the victims or beneficiaries and providers of these services have not been investigated within this context. Meanwhile, understanding these issues inform policy and practice aimed at enhancing PMTCT efforts in Ghana [20,21]. Therefore, this study sought to explore the impact of an inadequate healthcare infrastructure on the prevention of the mother-to-child transmission of HIV in Ghana by identifying key infrastructural barriers affecting PMTCT services, and examining the perceptions of healthcare providers and mothers regarding these barriers, with the aim of providing recommendations to improve the healthcare infrastructure for quality PMTCT services.

## 2. Theoretical Framework

### Health Systems Framework and the WHO Health Systems Building Blocks Model

This study utilises the Health Systems Framework, specifically focusing on the World Health Organization (WHO)’s Health Systems Building Blocks model, to examine infrastructural and resource barriers affecting the prevention of the mother-to-child transmission (PMTCT) of HIV in Ghana [22]. The WHO framework provides a structured approach for analysing key components of a healthcare system, especially in resource-limited settings, which is essential for understanding and addressing infrastructural barriers that hinder PMTCT services [23]. The model is made up of six essential components: service delivery, health workforce, health information systems, access to essential medicines, financing, and leadership and governance [24]. By applying this framework, the study identifies how deficiencies within these components affect the accessibility, quality, and confidentiality of PMTCT services in Ghana.

Thus, the model is used to explore the interconnected aspects of healthcare delivery and identify specific infrastructural issues impeding PMTCT services. For example, the service delivery and health workforce components are particularly relevant, as the study found that inadequate facilities and limited workforce support hinder the ability of healthcare providers to offer timely, confidential, and effective PMTCT services. Moreover, the ‘access to essential medicines’ component aligns with the findings on the lack of appropriate storage facilities for antiretroviral therapy (ART) medications, further compromising the effectiveness of PMTCT efforts [25,26].

The framework’s focus on health information systems also provides a critical understanding of how infrastructural deficiencies affect the documentation and continuity of PMTCT services. Meanwhile, limitations in the data systems or health information infrastructure can disrupt ART record-keeping, delay testing, and affect service tracking [27]. These issues highlight the relevance of the framework relating to the identification of specific infrastructural barriers to healthcare delivery concerning PMTCT, which is essential for creating actionable recommendations to strengthen the health system and reduce the mother-to-child transmission of HIV [24,28].

The application of the WHO Health Systems Framework in this qualitative study provides a comprehensive structure with which to analyse and interpret the data gathered from healthcare providers and mothers on their experiences with the relevant infrastructure and other resources concerning PMTCT services. The participants’ feedback in these areas enhanced the thematic analysis, aligning with the six components of the WHO model of health systems analysis. For instance, based on the issues identified in the service delivery and health workforce components, the study suggests increasing dedicated PMTCT spaces and improving staffing to enhance service quality. Similarly, addressing the access to essential medicines involves securing and improving ART storage facilities, ensuring that medications are safe and readily accessible for patients [25].

## 3. Materials and Methods

### 3.1. Study Area

The study was carried out at the Paga District Hospital and Chiana District Hospital of the Navrongo Municipality in the Upper East Region of Ghana. The Paga District Hospital faces infrastructural challenges, such as insufficient office space, inadequate storage for antiretroviral therapy (ART), and limited sanitation facilities, all of which can impact the delivery of PMTCT services. Similarly, Chiana District Hospital, located in the Chiana community, also caters to a rural population and experiences challenges that affect the quality of healthcare services [29]. The combination of these two hospitals for the study allows for a comprehensive examination of the infrastructural barriers affecting PMTCT services in a rural Ghanaian context [11].

This region is characterised by a predominantly rural population, with limited access to healthcare facilities and other healthcare resources [29]. The region has one of the highest rates of HIV prevalence in Ghana, making the effective implementation of prevention of mother-to-child transmission (PMTCT) services critical [30]. For instance, in 2019, the HIV prevalence among pregnant women in the region was reported to be 2.1%, with a prevalence rate among antenatal care attendees in Navrongo recorded at 2.0%. This highlights the importance of PMTCT services in preventing the transmission of HIV from infected mothers to their children [31].

The choice of this study area was influenced by the urgent need to address the infrastructural issues affecting PMTCT services in rural settings, particularly in light of the region’s high HIV prevalence rate of PMTCT, at 2.4%. Using these hospitals as cases in point, the research aims to provide valuable insights into how specific infrastructural barriers, such as inadequate facilities and logistical challenges, impact the accessibility and quality of PMTCT services for HIV-positive mothers. The rural nature of the Upper East Region poses additional challenges for healthcare access, including long travel distances to healthcare facilities, lack of public transportation, and poor road conditions [32,33]. These factors significantly affect the ability of HIV-positive mothers to access essential PMTCT services, highlighting the importance of addressing infrastructural barriers in improving health outcomes in this vulnerable population [34].

### 3.2. Study Design

The case study design was used to explore infrastructure-related barriers affecting the prevention of mother-to-child transmission (PMTCT) of HIV in Paga and Chiana Districts Hospitals in Navrongo, the Upper East Region [35]. This approach enables an in-depth examination of how the unique infrastructural and operational conditions within these hospitals affect PMTCT services, providing valuable insights that are specific to this rural setting [36]. The design further enhances a better understanding of how infrastructural issues and other healthcare resources, such as lack of office space, inadequate ART storage, and overcrowded outpatient departments (OPDs), impact service delivery [37].

### 3.3. Study Population and Participant Selection Criteria

According to the Ghana Statistical Service, the Paga District Hospital serves a catchment population of approximately 30,000 to 50,000 patients, while the Chiana District Hospital serves between 60,000 to 100,000 patients [38,39,40]. The study employed purposive sampling to select a total of 15 participants. These included healthcare providers and HIV-positive mothers who were actively accessing prevention of mother-to-child transmission (PMTCT) services at both hospitals. Participants were selected based on their direct involvement or experience with PMTCT services, and data collection continued until thematic saturation was achieved. This clarification ensures that the target population and participant selection process are explicitly defined. Purposive sampling was chosen because the study participants were deemed to have the requisite information regarding the subject matter under investigation and could provide rich and relevant insights into the study [41]. Eligible healthcare providers who were those actively involved in delivering PMTCT services at the Paga and Chiana District Hospitals. Moreover, the participants of the study were required to have a minimum of six months of experience in their respective roles. This was to ensure that the participants were familiar with the protocols and challenges associated with PMTCT services [42]. To obtain diverse perspectives on infrastructural and service delivery challenges encountered by the study facilities, medical doctors, nurses, and counsellors were interviewed. Similarly, the criteria for selecting HIV-positive mothers were designed to ensure that participants had sufficient engagement with PMTCT services to provide meaningful insights. Eligible mothers had to be currently receiving PMTCT services at either hospital and should have participated in the programme for at least three months. This timeframe allowed the mothers to develop a comprehensive understanding of the services provided and the infrastructural challenges associated with their care. Furthermore, willingness to share personal experiences concerning the healthcare facilities’ infrastructure was essential, as the study aimed to gather in-depth qualitative data from the mothers’ perspectives.

### 3.4. Data Collection Method, Tool, and Procedures

The study employed interviews as the primary data collection method, utilising an in-depth interview guide as the specific tool for gathering data, in addition to field notes during the data collection. The interview guide was piloted with similar hospitals to ensure that the questions were clear, relevant, and appropriately targeted to the study objectives. The pilot study was conducted with five participants who were not part of the main sample. All sessions took place face-to-face at a different facility, War Memorial Hospital. Participants were recruited through purposive sampling. Before participation, the aims of the study were explained, and participants were informed that their feedback would be used to refine the study procedures, but their data would not be included in the final analysis. The pilot helped ensure the clarity of study materials and the feasibility of the procedures. This approach was chosen to facilitate the participants’ experiences and perceptions regarding the infrastructural barriers affecting prevention of mother-to-child transmission (PMTCT) services in the selected rural district hospitals. In-depth interviews are particularly effective in qualitative research because they allow participants to express their thoughts and feelings in their own words, providing rich information and contextual insights into infrastructural challenges relating to the prevention of mother-to-child transmission of HIV [43].

The interviews were primarily conducted by the principal investigator, who is trained in qualitative methods (the first author, male), with assistance from the second author (male), who supported note-taking and logistical coordination to help reduce interviewer bias and ensure consistency across sessions among healthcare providers and HIV-positive mothers with the aim of obtaining their perceptions and experience of the challenges relating to lack of office space, inadequate ART storage, and limited sanitation facilities. There were no people present besides the participants and researchers. The participants knew about the researchers’ personal goals and reasons for performing the research, and the data were collected in a single session at the health facilities. This kind of interview allowed for flexibility in the questioning, thus enabling the interviewer to probe for clarity and emphasis [42]. The use of in-depth interviews directly aligned with the study objectives, which aimed to identify and understand the specific infrastructural barriers to PMTCT services in rural settings. This qualitative data provided the foundation for identifying actionable recommendations to improve infrastructure and, consequently, health outcomes for HIV-positive mothers and their infants [42]. All interviews were audio-recorded and lasted between 40–60 min, reaching the point of data saturation at the 15th person.

### 3.5. Data Analysis

The software NVivo 12 was used to manage and organise the qualitative data first. The data was analysed following the framework established by Braun and Clarke’s six phases of thematic analysis in qualitative inquiry [44,45]. The voice recordings of the interviews conducted were transcribed verbatim. To understand these recordings, the first phase of Braun and Clarke’s thematic analysis was employed, where the data analyst had to familiarise themselves with the data. This process of familiarisation was critical, as it enabled the researchers to immerse themselves in the participants’ narratives and identify initial themes and patterns within the data. Following this, the researchers generate an initial 20 codes from the transcripts, focusing on significant segments related to the study’s objectives. These codes are to highlight the various infrastructural challenges, such as inadequate facilities, insufficient logistics, and their effect on the care provided to HIV-positive mothers [46]. The authors helped describe the coding tree.

After coding, the initial codes were re-grouped into broader themes that represented recurring patterns across the dataset. This leads to reviewing and refining the themes to ensure accurate representation of the data as shared by participants of the study. The themes were derived from the data. The final stage of the thematic analysis involved defining and naming the identified themes, which helped to clarify the significance of the study objectives. Each theme was illustrated with direct quotes from participants, providing concrete evidence and enhancing the credibility of the findings. Employing Braun and Clarke’s thematic analysis, the study derived rich insights into the specific infrastructural and resource factors affecting PMTCT services in rural district hospitals, ultimately informing strategies for improvement in healthcare delivery for HIV-positive mothers. After the analysis, the transcripts were returned to participants for comments and corrections. The participants were also provided feedback on the findings.

## 4. Ethical Considerations

This study was conducted following the principles outlined in the Declaration of Helsinki (1975, revised in 2013). Ethical clearance for the study was obtained from the Ghana Health Service (GHS) on 7 December 2021, with approval code GHS/UE/RS/11/001, ensuring that the research complied with the national ethical guidelines and standards for research involving human subjects. Before participation, informed consent was obtained from all participants.

## 5. Results

The findings of the study are presented in major thematic areas, including the socio-demographic features of the participants of the study and the major themes emerging from the findings of the study. These include infrastructure and facility limitations, logistical challenges, lack of privacy and confidentiality, and human resource challenges.

### 5.1. Socio-Demographic Characteristics of Participants

A total of 15 participants were involved in the study, comprising 7 healthcare providers and 8 mothers seeking PMTCT (Prevention of Mother-to-Child Transmission) services. The majority were female (12), with 3 males. Most participants were between 18 and 40 years old, with 5 aged 18–30 and 6 aged 31–40.

In terms of education, 6 participants had secondary education, 4 had tertiary education, 4 had primary education, and 1 had no formal education. Among healthcare providers, occupations included 2 doctors, 3 nurses, 1 pharmacist, and 1 midwife. For mothers, 4 had one child, 2 had two children, and 2 had three or more children. All 8 mothers were HIV-positive and receiving PMTCT services as also indicated in the Table 1 below.

### 5.2. Infrastructure and Facility Limitations

This theme includes all aspects of the physical environment that directly affect the delivery of PMTCT services. Under this theme emerged three sub-themes explaining how the issues of infrastructure or healthcare facilities affect the quality of PMTCT services in the study areas.

#### 5.2.1. Lack of Dedicated Office Spaces

The findings indicate that the majority of the participants—approximately 80%—reported that the lack of private office spaces significantly limits healthcare providers’ ability to conduct consultations and manage patient care effectively. This sub-theme highlights a critical infrastructural barrier within many healthcare facilities, particularly in resource-constrained environments or high-volume clinical settings.

*“Without a private space, it’s difficult to have sensitive discussions with patients. It affects both confidentiality and the quality of care we provide.”*

(Healthcare Provider)

*“We are forced to manage consultations in shared areas, which makes it hard to focus and offer patients the attention they deserve.”*

(Healthcare Worker)

*“Patients hesitate to open up when they know others can hear. A proper office space is not a luxury; it’s a necessity for effective care.”*

(Medical Officer)

#### 5.2.2. Limited Outpatient Departments (OPDs)

Insufficient OPD facilities designated for PMTCT patients hinder timely service provision, leading to overcrowded waiting areas. This congestion affects patient comfort, privacy, and overall service efficiency. Delays in accessing care may compromise the effectiveness of PMTCT interventions. The overcrowding also increases the risk of cross-infection among vulnerable patients. Healthcare workers reported an increased workload and difficulty managing patient flow under these conditions.

*“The OPD is always overcrowded. We struggle to accommodate all the patients, leading to long wait times and frustration.”*

(Healthcare Worker)

*“There aren’t enough consultation rooms, so some patients are forced to wait for hours just to be seen.”*

(Nurse)

*“Expecting mothers seeking PMTCT services have to wait in the general outpatient queue, which delays care and increases their stress.”*

(Midwife)

#### 5.2.3. Inadequate Washrooms

Participants reported that the lack of clean and accessible washroom facilities negatively affects patient comfort and basic hygiene. This issue is especially concerning for mothers seeking care, who may need frequent restroom access during long clinic visits. Poor sanitation can deter patients from returning for follow-up appointments. It may also pose health risks, particularly in maternal and child health settings. The inadequacy reflects broader infrastructure gaps impacting service quality.

*“Patients, especially pregnant mothers, complain about the lack of clean washrooms. It’s a basic necessity that we struggle to provide.”*

(Health Facility Staff)

*“There are too few toilets for the number of patients we see daily. Many are in poor condition, making it uncomfortable for everyone.”*

(Clinic Administrator)

*“It’s difficult to manage hygiene properly when washrooms are either inaccessible or unhygienic. This impacts both staff and patients.”*

(Nurse)

### 5.3. Logistical Challenges

This theme equally explored issues relating to some healthcare resources, such as logistics, which support the provision of PMTCT services in the rural District Hospital of the Upper East Region.

#### 5.3.1. Inadequate Facilities for Storing ART Medications

The findings highlighted a lack of proper storage facilities for antiretroviral therapy (ART) medications, affecting their efficacy and safety. Participants noted issues such as poor temperature control, limited shelving, and a lack of secure storage. These conditions can lead to the medication becoming spoiled or stock mismanagement. Inadequate storage also complicates inventory tracking and timely dispensing. Ultimately, this jeopardises consistent access to life-saving treatment for patients.

*“Without proper storage facilities, we cannot guarantee the safety and efficacy of the ART medications we provide.”*

(Pharmacist)

*“The lack of secure, temperature-controlled storage makes it difficult to ensure that patients receive medications that are still effective.”*

(Healthcare Provider)

*“Improper storage of ART medications puts both patients and healthcare providers at risk, compromising treatment outcomes.”*

(Clinic Manager)

#### 5.3.2. Shortage of Testing Equipment

Participants reported that insufficient testing equipment limits the ability to diagnose and monitor HIV effectively. This shortage results in delayed test results and prolonged waiting times for patients. It also hampers the timely initiation or adjustment of treatment plans. The lack of essential diagnostic tools undermines the quality of HIV care. Healthcare workers expressed frustration over their inability to provide prompt, accurate services.

*“We often have to wait for equipment to become available, which causes delays in diagnosing and monitoring HIV. This is unacceptable when timely intervention is critical.”*

(Laboratory Technician)

*“Limited testing equipment means that we can’t perform necessary tests on all patients, and that delays the start of treatment or adjustments to care.”*

(Medical Officer)

*“The shortage of testing equipment makes it difficult to provide the standard of care expected. It’s a constant challenge, especially in high-demand areas.”*

(Nurse)

### 5.4. Lack of Privacy and Confidentiality

This theme encompasses issues that affect the privacy and confidentiality of patients within the healthcare setting, as indicated by approximately 80% of participants.

#### 5.4.1. Overcrowded OPDs

The study findings reveal that mothers expressed concern over overcrowding in outpatient departments. This situation compromises confidentiality, as sensitive discussions can be overheard by others. The lack of privacy discourages open communication between patients and providers. Overcrowding also contributes to long waiting times and increased stress. Such conditions hinder the delivery of respectful and patient-centred care.

*“It’s uncomfortable to talk about personal health issues when others are around. The overcrowded OPD compromises my confidentiality.”*

(Mother/Patient)

*“I feel like I have no privacy when I go to the OPD. Sensitive matters are discussed in full view of others, and that’s not okay.”*

(Patient)

*“Overcrowded waiting areas make it difficult to feel like I’m being treated with dignity. I don’t want others to overhear my health concerns.”*

(Mother Seeking Care)

#### 5.4.2. Limited Spaces Impacting Comfort

The lack of adequate private spaces for consultations leaves patients feeling exposed and uncomfortable. This environment discourages them from sharing personal or sensitive health information. Patients, especially mothers, fear being overheard during discussions. Such limitations reduce trust in the healthcare system.

*“The lack of a private space for consultations makes me feel vulnerable. I’m hesitant to share important details about my health.”*

(Patient)

*“Having to discuss personal health matters in a public space feels disrespectful. I need a quiet, private setting to feel safe.”*

(Mother)

*“I worry that my personal health information isn’t safe when there’s no private space for consultations. It affects how open I am with my healthcare provider.”*

(Patient)

### 5.5. Human Resource Challenges

This theme captures how infrastructural barriers affect the overall quality of care provided to patients, with particular reference to PMTCT services.

#### 5.5.1. Inability to Provide Timely Services

Healthcare providers reported that infrastructural limitations delay the delivery of PMTCT services. Issues such as limited consultation rooms, inadequate equipment, and overcrowded facilities slow down service provision. These delays can lead to missed opportunities for early intervention. Timeliness is critical in preventing mother-to-child HIV transmission. Consequently, patient outcomes and programme effectiveness are negatively affected.

*“Inadequate infrastructure means we cannot provide the timely care that PMTCT patients desperately need. Delays directly impact outcomes.”*

(Healthcare Provider)

*“When facilities are limited, we can’t see patients quickly, which delays critical interventions for both mother and child.”*

(Medical Officer)

*“The lack of proper infrastructure prevents us from adhering to care schedules, and this compromises the effectiveness of our services.”*

(Nurse)

#### 5.5.2. Increased Stress and Anxiety

The study identified that inadequate facilities contribute to heightened stress and anxiety among mothers seeking care. Discomfort and a lack of privacy during consultations make them feel exposed and uneasy. This emotional strain can deter them from returning for follow-up visits. Fear of judgment or being overheard discourages open communication. As a result, their willingness to seek timely and consistent care is affected.

*“The discomfort of waiting in crowded spaces, without privacy, increases my anxiety. I’m often too stressed to even focus on the care I need.”*

(Mother Seeking PMTCT Care)

*“The lack of private spaces makes me feel exposed and uncomfortable. This anxiety makes it harder for me to take the necessary steps in my care.”*

(Patient)

*“The stress of dealing with uncomfortable facilities makes it harder for me to keep my appointments, and I often feel too overwhelmed to ask the questions I need to.”*

(Mother)

#### 5.5.3. Limited Staff Support

An inadequate physical infrastructure restricts healthcare providers from offering private and professional interactions with patients. This limitation reduces the ability to provide emotional and informational support during visits. Without proper space, staff struggle to build trust and address patient concerns fully. The lack of privacy also hampers effective counselling and follow-up. Consequently, patients receive less comprehensive care and feel less supported.

*“We are stretched too thin due to the lack of proper space and support. This affects our ability to give each patient the care and attention they deserve.”*

(Healthcare Provider)

*“Limited infrastructure leads to burnout. We have fewer resources and staff, and as a result, we can’t provide the personalized support that patients need.”*

(Nurse)

*“When we are understaffed and the facilities are inadequate, we can’t be as present for our patients, which impacts the quality of care we provide.”*

(Medical Officer)

## 6. Discussion

This study highlights the critical structural and systemic barriers affecting the implementation of PMTCT services in Ghana, including infrastructure deficits, workforce shortages, and supply chain challenges. Beyond these operational constraints, the findings reveal a disconnect between national priority setting, benefits package design, and the realities of healthcare delivery on the ground. While global and national models emphasise cost-effective interventions, their impact is undermined when health systems lack the capacity to implement them. Framed within the WHO model, this discussion explores how these challenges affect PMTCT service delivery and calls for a more context-responsive approach to health policy, one that integrates system-strengthening efforts like the StEPS initiative with grounded, qualitative insights to inform policy and programme design.

### 6.1. Infrastructure and Facility Limitations

The lack of dedicated office spaces, overcrowded outpatient departments (OPDs), and inadequate washroom facilities significantly impact the quality of prevention of mother-to-child transmission (PMTCT) services. According to the WHO Health Systems Model, health service delivery should be designed to ensure accessibility, effectiveness, and patient-centred care [47,48]. However, the findings indicate that the absence of private office spaces compromises confidentiality and the quality of care, contradicting the best practices outlined in the existing literature [8,22,47].

Crowding in OPDs is another critical challenge. Research suggests that high patient volumes in poorly designed health facilities lead to longer wait times, increased patient frustration, and reduced provider efficiency [10,49]. Furthermore, limited and unhygienic washroom facilities undermine patient dignity and hygiene, reinforcing the inequities in maternal healthcare. The Health Systems Framework also highlights the importance of a well-functioning infrastructure in ensuring positive health outcomes; yet, these structural deficiencies limit service effectiveness [12,50].

This study sheds light not only on the structural and resource constraints hampering the PMTCT efforts in Ghana but also highlights a critical disconnect between priority setting, benefits package design, and the realities on the ground. While national models and global guidelines may identify cost-effective interventions, the effectiveness of these interventions is contingent on the strength of the health system delivering them.

### 6.2. Logistic Challenges

Logistical issues, including inadequate storage for antiretroviral therapy (ART) medications and a shortage of HIV testing equipment, impede the delivery of PMTCT services. Proper medication storage is crucial for maintaining drug efficacy, as ART requires temperature-controlled environments to prevent degradation [8,51]. The WHO [8,52] guidelines emphasise the need for reliable supply chain management in order to ensure drug safety and accessibility. However, the findings highlight the challenges in maintaining adequate facilities, consistent with previous studies that have documented similar barriers in resource-limited settings [13,53].

A limited access to HIV testing equipment further exacerbates delays in diagnosis and treatment initiation, which is essential for effective PMTCT programmes. The WHO Health Systems Model underscores the importance of medical products and technologies in enhancing health service performance [14,22,24]. The shortage of diagnostic tools, therefore, undermines timely interventions and patient outcomes, as observed in a study focusing on healthcare challenges in sub-Saharan Africa [18,53].

The findings underscore that healthcare infrastructure gaps, workforce shortages, and supply chain failures directly undermine the implementation of PMTCT services, rendering even the most efficient or “cost-effective” strategies impractical in real-world settings.

### 6.3. Lack of Privacy and Confidentiality

Privacy concerns in healthcare settings significantly impact patient willingness to seek and adhere to treatment. The findings indicate that overcrowding and a lack of designated private spaces for consultations compromise confidentiality, a fundamental component of patient rights [19,54]. Confidentiality issues have been identified as barriers to HIV care uptake, as patients fear stigma and discrimination when discussing sensitive health issues in public settings [10,55].

The WHO Health Systems Framework also identifies responsive and people-centred services as a cornerstone of quality healthcare. The lack of privacy contradicts this principle, highlighting the need for structural improvements to foster trust and engagement in PMTCT programmes. Studies suggest that private consultation areas can enhance patient–provider communication and improve the adherence to ART and follow-up care [23,56].

This reinforces the need for health system reforms that go beyond infrastructure to ensure that PMTCT service environments support confidentiality, dignity, and trust, critical for effective prevention and treatment adherence.

### 6.4. Human Resource Challenges

Infrastructural deficiencies place a heavy burden on healthcare workers, leading to delays in service provision, increased stress, and staff burnout. The Health Systems Framework highlights human resources as a critical component of effective healthcare delivery [21,24]. However, an inadequate physical infrastructure and high patient volumes contribute to staff fatigue, limiting the quality of care provided. Research has shown that poor working conditions negatively affect healthcare workers’ performance and motivation, ultimately impacting patient outcomes [23,57].

Additionally, stress and anxiety among mothers seeking PMTCT services are exacerbated by overcrowded and uncomfortable facilities. Studies indicate that a supportive healthcare environment can improve patient engagement and adherence to care [27,58]. Without an adequate infrastructure and staff support, patient trust in the healthcare system diminishes, leading to suboptimal health outcomes for mothers and their infants.

Therefore, this study advocates for a more context-aware approach to health policy design, one that bridges the gap between health-system-strengthening efforts (such as the StEPS initiative) and the formulation of intervention priorities [22,59]. To ensure an impact, priority setting and benefits package design must be informed by the perception and perspectives from participants, accounting for infrastructure limitations, human resource constraints, and systemic bottlenecks. Aligning intervention strategies with real-world constraints will be essential to improving the reach and sustainability of PMTCT programmes in Ghana [26,60].

## 7. Conclusions

This study highlights that the effective prevention of the mother-to-child transmission (PMTCT) of HIV in Ghana is not solely a matter of adopting cost-effective interventions, but rather hinges on the health system’s capacity to deliver these services reliably and equitably [61]. Persistent infrastructural deficits, human resource shortages, and logistical constraints can significantly hinder the quality and accessibility of PMTCT services, compromising confidentiality, continuity of care, and timely intervention.

Moreover, the findings reveal a critical disconnect between national priority setting, benefits package design, and the lived realities within healthcare settings. While global and national frameworks promote evidence-based, cost-efficient strategies, their successful implementation requires a responsive and resilient health system [62].

To enhance PMTCT outcomes, health policies must be informed by grounded, context-specific evidence that reflects the operational realities of healthcare workers and clients. Bridging the gap between policy design and delivery through integrated health-system-strengthening efforts, such as the StEPS initiative, is essential for improving maternal and child health outcomes in Ghana and similar low-resource settings. A more holistic and inclusive approach to health planning, rooted in qualitative insights from the ground, is critical for ensuring that PMTCT interventions are not only technically sound but also practically feasible and sustainable [63].

### 7.1. Limitations, Policy, and Practice in PMTCT Delivery

Firstly, the study primarily relies on qualitative data, which, although rich in detail, may not fully capture the breadth of the issues across different healthcare settings. Data for this study were also collected in 2022, and, while the findings continue to reflect the persistent challenges in PMTCT service delivery, some contextual factors may have evolved since the time of data collection. The perspectives shared by healthcare providers and patients may not be universally representative, as they are based on a specific context or geographical location.

Moreover, the study does not account for the potential impact of external factors such as governmental policies, funding limitations, or broader healthcare system challenges, which could also influence the delivery and quality of PMTCT services. Future research could address these limitations by incorporating quantitative data, exploring the longitudinal impacts, and considering external systemic influences on healthcare delivery.

The qualitative insights from this study reveal that healthcare providers are often forced to navigate broken or inefficient systems to deliver critical PMTCT services. This may not only affect the service quality and coverage but also contribute to healthcare worker fatigue and client mistrust. The gap between what is outlined in policy and what is achievable in practice is particularly stark in rural and under-resourced facilities [64].

To bridge this divide, there is a need for iterative, bottom-up feedback loops between service delivery points and policymakers. Qualitative evidence like that presented in this study should inform national priority setting, ensuring that the chosen interventions are not only theoretically cost-effective but also practically implementable within the existing system constraints.

Embedding PMTCT delivery into the broader health system strengthening agenda will improve coordination, resource allocation, and accountability. Ultimately, this approach can enhance the impact of PMTCT programmes and support Ghana’s broader goal of eliminating the mother-to-child transmission of HIV [65].

### 7.2. Implications for Policy and Health Systems Strengthening

The findings from this study highlight the persistent infrastructural and resource-related barriers that continue to undermine the effective delivery of PMTCT services in Ghana. These challenges include the limited availability of testing supplies, inconsistent ART (antiretroviral therapy) stock, understaffing, and inadequate training for healthcare workers, challenges echoed in prior studies across sub-Saharan Africa [66,67]. While these issues are well-documented, their persistence raises critical questions about how national health priorities are set and how benefits packages are designed without sufficiently accounting for on-the-ground realities.

At the policy level, cost-effective interventions for PMTCT are frequently proposed through models and global evidence-based frameworks [68,69]. However, the implementation of these interventions is often constrained by the underlying health system’s capacity. This disconnect suggests that current priority-setting mechanisms may be overly focused on theoretical efficiency rather than practical deliverability [61]. Without concurrent investments in infrastructure, human resources, and health systems strengthening, even the most well-designed interventions risk falling short in practice [70,71].

Initiatives like Ghana’s StEPS (Systems for Health), which aims to integrate service delivery with systems strengthening, offer an opportunity to realign these efforts [59]. By linking intervention design with systems-level reform, there is potential to address the structural weaknesses that inhibit service delivery. Our findings support the integration of PMTCT goals into broader health systems strategies, rather than treating them as standalone or vertical programmes. This systems-thinking approach is increasingly recognised as essential for long-term sustainability and effectiveness [60,70,71].

## Figures and Tables

**Table 1:** Socio-demographic Characteristics of Study Participants (N = 15).

Characteristic	Category	Frequency (N = 15)
Type of Participant	Healthcare providers	7
	Mothers (seeking PMTCT)	8
Gender	Male	3
	Female	12
Age Group	18–30 years	5
	31–40 years	6
	41–50 years	3
	51 years and above	1
Educational Level	No formal education	1
	Primary education	4
	Secondary education	6
	Tertiary education	4
Occupation (Healthcare Providers)	Doctor	2
	Nurse	3
	Pharmacist	1
	Midwife	1
Number of Children (Mothers)	1 child	4
	2 children	2
	3 or more children	2
HIV Status (Mothers)	HIV-positive and accessing PMTCT services	8

Source: Field data (2022).

## Data Availability

No data is available for public access as it is confidential and contains sensitive information regarding participants.
